# A High-Tryptophan Diet Reduces Seizure-Induced Respiratory Arrest and Alters the Gut Microbiota in DBA/1 Mice

**DOI:** 10.3389/fneur.2021.762323

**Published:** 2021-11-23

**Authors:** Qiang Yue, Mingfei Cai, Bo Xiao, Qiong Zhan, Chang Zeng

**Affiliations:** ^1^Department of Neurology, Xiangya Hospital, Central South University, Changsha, China; ^2^Department of Neurology, The Second Xiangya Hospital, Central South University, Changsha, China; ^3^Health Management Center, Xiangya Hospital, Central South University, Changsha, China

**Keywords:** SUDEP, high-tryptophan diet, 5-HT, gut microbiota, DBA/1 mice

## Abstract

**Background and Aims:** Central 5-hydroxytryptamine (5-HT) defects are responsible for the occurrence of sudden unexpected death in epilepsy (SUDEP). The DBA/1 mouse is an animal model of SUDEP since the mouse exhibits audiogenic seizure-induced respiratory arrest (S-IRA). The synthesis of central 5-HT is closely related to the gut microbiota. Moreover, emerging studies suggest a possible role for the microbiota in mitigating seizure likelihood. Based on this, we aimed to explore the effect of a high-tryptophan diet (HTD) on SUDEP as well as the synthesis and metabolism of central 5-HT. Furthermore, we investigated the involvement of the gut microbiota in this process.

**Methods:** All DBA/1 mice were subjected to acoustic stimulation to induce seizures. Only those mice that exhibited S-IRA were randomly assigned to the normal diet (ND) group (*n* = 39) or HTD group (*n* = 53). After 1 month of dietary intervention, (1) S-IRA rates were evaluated, (2) the concentrations of 5-HT and its metabolite 5-hydroxyindoleacetic acid (5-HIAA) in the plasma and brain were determined by ultra-high-pressure liquid chromatography, and (3) the fecal flora biodiversity and species composition were analyzed by 16S rDNA microbiota profiling.

**Results:** The S-IRA rate in DBA/1 mice was significantly reduced in the HTD group compared with that in the control group. HTD increased the levels of 5-HT and 5-HIAA in both the telencephalon and midbrain. HTD significantly elevated the species richness and diversity of the gut microbiota. Moreover, there was a significant difference in the gut microbiota composition between the two groups, and the intestinal flora was dominated by *Proteobacteria* and *Actinobacteria* after HTD.

**Conclusions:** HTD is efficient in lowering S-IRA rates and elevating the central 5-HT level in DBA/1 mice. The gut microbiota was altered after HTD intervention. The significant increase in *Proteobacteria* and *Actinobacteria* may be related to the SUDEP-protective effect of HTD. Our findings shed light on a candidate choice of dietary prevention for SUDEP.

## Introduction

Sudden unexpected death in epilepsy (SUDEP) is one of the main causes of death in patients with epilepsy (1–3). It has become a common public health burden among neurological diseases (4). However, the pathogenesis of SUDEP remains elusive. It is generally believed that seizure-induced arousal as well as respiratory and cardiac dysfunction are major causes of SUDEP (2, 5). DBA/1 mice have been regarded as appropriate animal models for studying SUDEP. The mouse can present generalized audiogenic seizures (AGSz), followed by seizure-induced respiratory arrest (S-IRA) and sudden death induced by acoustic stimulation. This is consistent with the respiratory dysfunction most often witnessed in patients with SUDEP (6, 7). The susceptibility to AGSz in DBA/1 mice remains high on postnatal day (PND) 100 (6). Evidence has shown that cardiac dysfunction in DBA/1 mice lagged behind S-IRA, and dying animals could be resuscitated by assisted ventilation (7). This suggests that S-IRA may be the main cause of death in this SUDEP model.

5-Hydroxytryptamine (5-HT) is an important neurotransmitter mainly synthesized in the nuclei of the midbrain and medullary raphe of the brainstem (8). 5-HT plays a key role in modulating arousal, respiratory, and cardiac functions by corresponding control centers in the upper and lower brainstems (8–10). In the brain, tryptophan hydroxylase 2 (TPH2) catalyzes tryptophan (TRP) to synthesize 5-hydroxytryptophan (5-HTP). 5-HTP is then decarboxylated by aromatic L-amino acid decarboxylase to form 5-HT. 5-Hydroxyindoleacetic acid (5-HIAA) is the end product of 5-HT metabolism (11). Our previous research found that the 5-HTP, 5-HT, and 5-HIAA contents and TPH2 activity in the brainstem of DBA/1 mice were significantly decreased compared with those in the brainstem of C57BL/6 mice (12). The intraperitoneal injection of fluoxetine, a selective 5-HT reuptake inhibitor (SSRI), or 5-HTP, the precursor of 5-HT, significantly reduced the incidence of S-IRA in DBA/1 mice (13, 14). In contrast, the incidence of S-IRA in DBA/1 mice markedly increased after pretreatment with 5-HT antagonists (15, 16). The above evidence suggests that central 5-HT deficiency is probably an underlying reason for SUDEP in DBA/1 mice.

The synthesis of 5-HT is closely related to the gut microbiota (17). Almost 90% of 5-HT in the human body is produced in the gut by enterochromaffin cells (18, 19). Furthermore, the intestinal flora may be connected with the central nervous system (CNS) through the dynamic two-way “gut-brain axis” (20) and can affect brain function (21) and central neurotransmitters, including 5-HT (22). The intestinal flora is therefore involved in neurological diseases (23). Previous studies demonstrated that the concentrations of 5-HT and 5-HIAA in the hippocampus of male germ-free (GF) animals were significantly higher than those of control animals (24). Supplementation with probiotics largely increased the level of central 5-HT in animals (25, 26). The above evidence indicates that the gut microbiota mediates the synthesis and metabolism of peripheral and central 5-HT.

Mounting evidence suggests that the gut microbiota mediates seizure susceptibility. Studies have demonstrated that probiotics have a protective effect on sensitivity to anti-seizure drugs in patients with drug-resistant epilepsy (27, 28). Moreover, a retrospective study reported that six patients with drug-resistant epilepsy achieved temporary seizure freedom during antibiotic treatment (29). An animal study also found that the gut microbiota was required for protection against acute electrically stimulated seizures and spontaneous tonic-clonic seizures caused by a ketogenic diet (KD) (30).

As an important essential amino acid for 5-HT synthesis, TRP is mainly obtained from the diet (31). The oral administration of TRP or chronic high-tryptophan diet (HTD) intervention in rats can largely increase 5-HT levels in the CNS (32, 33). Based on the above evidence, we hypothesized that HTD could reduce the occurrence of SUDEP in DBA/1 mice. Moreover, our study aimed to explore how 5-HT and its metabolite changed in the plasma and brain and whether the gut microbiota was altered during the process.

## Materials and Methods

### Animals

The study complied with the guidelines of the Care and Use of Laboratory Animals (NIH USA), and experimental protocols were approved by the Animal Ethical and Welfare Committee and the Institutional Animal Care and Use Committee, Xiangya Hospital, Central South University, China (No. 202009559). Wild-type male DBA/1 mice were obtained from the Hunan SJA Laboratory Animal Co., Changsha, Hunan, China. All DBA/1 mice were housed five to six/cage in a standard animal facility under controlled conditions (temperature 22 ± 3°C, humidity 55 ± 5%) with a 12 h light-dark cycle and had free access to food and water. All efforts were made to reduce the number of animals used and their suffering.

### Seizure Induction and Resuscitation

All DBA/1 mice (from PND 26–28) were subjected to an acoustic stimulation paradigm and induced daily for three consecutive days (each interval was more than 24 h) to evoke AGSz and S-IRA (6, 12). Briefly, each mouse was placed in a transparent plastic chamber and stimulated continuously with a 110 dB electric bell (Zhejiang People's Electronics, Zhejiang, China) for 60 seconds or until the mouse exhibited tonic seizures. DBA/1 mice with S-IRA were resuscitated by an animal ventilator. S-IRA was defined as the cessation of movement of the chest, which would lead directly to the animal's death unless resuscitation was instituted within 5–6 s. Since the seizure occurrence is easy to detect by behavior (typically exhibit as wild running, generalized clonus, and tonus, ending in tonic hind limb extension in most cases), EEG or other methodology was not used to detect seizure occurrence in this study. DBA/1 mice with at least one S-IRA were considered successfully primed. Only primed DBA/1 mice were used in subsequent experiments.

### Experimental Design and Grouping

The primed animals were randomly assigned to the normal diet (ND) group (*n* = 39) or HTD group (*n* = 53) for a 1-month dietary intervention. The animals in the ND group were fed a ND with a TRP level of 2.1 g/kg, while the TRP level of the HTD group was increased to 4 g/kg, as previously reported (34) (Supplementary Tables 1, 2). Both diets were prepared by the Beijing Keao Xieli Feed Co., Beijing, China. After 1 month of dietary intervention, acoustic stimulation was reapplied in both groups. Then, the S-IRA rates, levels of 5-HT and 5-HIAA, and composition and diversity of intestinal flora were examined in both groups (Supplementary Figure 1).

### 16S rDNA Microbiota Profiling

The animals were placed into a clean cage lined with sterile filter paper. Fecal samples were collected immediately after defecation, quick-frozen in liquid nitrogen, and stored at −80°C. Microbial community genomic DNA was extracted from fecal samples using the E.Z.N.A.^®^ DNA Kit (Omega Bio-tek, Norcross, GA, U.S.) according to manufacturer's instructions. The DNA extract was checked on a 1% agarose gel, and the DNA concentration and purity were determined with a NanoDrop 2000 UV-vis spectrophotometer (Thermo Scientific, Wilmington, USA). The hypervariable V3–V4 region of the bacterial 16S rRNA gene was amplified with the primer pairs 338 F (5′-ACT CCT ACG GGA GGC AGC AG-3′) and 806 R (5′-GGA CTA CHV GGG TWT CTA AT-3′) (35) by a PCR thermocycler (ABI GeneAmp^®^ 9700, CA, USA). PCR amplification of the 16S rRNA gene was performed as follows: initial denaturation at 95°C for 3 min, followed by 27 cycles of denaturing at 95°C for 30 s, annealing at 55°C for 30 s and extension at 72°C for 45 s, a single extension at 72°C for 10 min, and a final extension at 4°C. The PCR mixtures contained 5 ×4 μL of *TransStart* FastPfu buffer, 2 μL of 2.5 mM dNTPs, 0.8 μL of forward primer (5 μM), 0.8 μL of reverse primer (5 μM), 0.4 μL of *TransStart* FastPfu DNA polymerase, 10 ng of template DNA, and ddH_2_O up to 20 μL. The PCRs were performed in triplicate. The PCR product was extracted from a 2% agarose gel and purified using the AxyPrep DNA Gel Extraction Kit (Axygen Biosciences, Union City, CA, USA) according to the manufacturer's instructions and quantified using a Quantus™ Fluorometer (Promega, USA). Purified amplicons were pooled in equimolar amounts and paired-end sequenced (2 ×300) on an Illumina MiSeq platform (Illumina, San Diego, USA) according to standard protocols. The raw 16S rRNA gene sequencing reads were demultiplexed, quality-filtered by Trimmomatic, and merged by FLASH. Operational taxonomic units (OTUs) with a 97% similarity cutoff (36) were clustered using UPARSE software (Uparse v7.0.1001), and chimeric sequences were identified and removed. The taxonomy of each OTU representative sequence was analyzed by RDP Classifier against the 16S rRNA database using a confidence threshold of 0.7. The metagenomic analysis of intestinal flora was analyzed on the Majorbio I-Sanger Cloud Platform (www.i-sanger.com). Additionally, alpha diversity was analyzed using the Chao estimator (an index of species relative abundance), observed richness (Sobs), and Shannon diversity index (an index of the complexity of species diversity) to reflect species diversity and richness. Beta diversity analysis was calculated through cluster tree analysis to study the similarities or differences in community structures among different samples, and principal coordinate analysis (PCoA) was used to compare group differences in the overall microbiota profile.

### Ultra-High-Pressure Liquid Chromatography

The animals were intraperitoneally injected with 1% chloral hydrate (400 mg/kg) for deep anesthesia. Cardiac blood (0.5–1.0 mL) was carefully extracted from each mouse. Then, the mouse was killed by decapitation for the collection of brain tissue. A mark was made in the anterior fontanel as the bregma point. The brain tissue was cut at bregma −3 mm, −5.5 mm, and −9 mm and carefully separated on ice. Blood and brain samples were rapidly frozen in liquid nitrogen and stored at −80°C away from light. The contents of 5-HT and 5-HIAA of each sample were quantified on an ultra-high-pressure liquid chromatography (UHPLC)-MS/MS platform. The compounds 5-HT and 5-HIAA were labeled with benzoyl-13C6 chloride and used as internal standards for quantification. All analytical standards and internal standards were prepared individually at a concentration of 1 mg/mL as a stock solution. The samples of calibration curves were finally obtained by mixing the calibration curve solution with internal standard solution (benzoyl-13C6 chloride-derivatized standard mixture) to generate calibration levels covering a range of 0.0,016–8 μM for 5-HT and 5-HIAA. UHPLC-MS/MS analysis was performed on an Agilent 1290 Infinity II UHPLC system coupled to 6470A Triple Quadrupole mass spectrometer (Santa Clara, CA, United States). The samples were injected onto a Waters UPLC BEH C18 column (100 mm ×2.1 mm, 1.7 μm) at a flow rate of 0.4 mL/min. The mobile phase consisted of water in 10 mmol/L ammonium formate and 0.15% formic acid (A) and acetonitrile (B). Chromatographic separation was conducted by a gradient elution program as follows: 0.5 min, 1% B; 1 min, 5% B; 4 min, 15% B; 6 min, 30% B; 7 min, 30% B; 7.5 min, 50% B; 9.5 min, 70% B; 9.6 min, 100% B; 10.6 min, 100% B; 10.7 min, 1% B; and 12.5 min, 1% B. The column temperature was 40°C. The eluted analytes were ionized in an electrospray ionization source in the positive mode (ESI+). The temperature of the ESI+ source drying gas was 300°C, and that of the sheath gas was 350°C. Dynamic multiple reaction monitoring (dMRM) was used to acquire data in the optimized MRM transition. The total scan time per cycle was 300 ms. Agilent MassHunter software (version B.08.00) was used to control instruments and acquire data. The raw data were processed by Agilent MassHunter Workstation Software (version B.08.00) using the default parameters. The peak areas of the target compounds were integrated, and the output was used for quantitative calculation.

### Statistics

Statistical analysis was performed using Statistical Product and Service Solutions 19.0 software. The incidence of S-IRA between the two groups was compared using the chi-square test. The concentrations of 5-HT and 5-HIAA between the two groups were compared using independent sample *t*-tests and expressed as the mean ± SD. For gut microbiota analysis, normally distributed data were compared using Student's *t*-test, and non-parametric data were compared using the Wilcoxon rank-sum test. Statistical significance was inferred if *p* < 0.05.

## Results

### HTD Reduced S-IRA Susceptibility and Modulated 5-HT Metabolism in DBA/1 Mice

**Figure 1:**
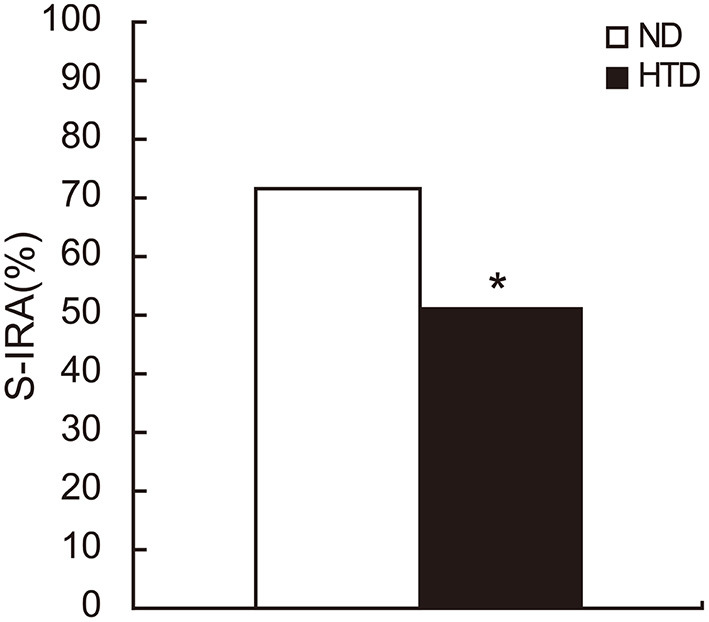
The S-IRA rate in DBA/1 mice was significantly decreased in the HTD group. ND, *n* = 39. HTD, *n* = 53. Statistical analysis was performed by the chi-square test. *p < 0.05. S-IRA, seizure-induced respiratory arrest; ND, normal diet; HTD, high-tryptophan diet.

The incidence of S-IRA was significantly lower in the HTD group than in the ND group (50.94% vs. 71.79%, *p* < 0.05) (Figure 1).

**Figure 2:**
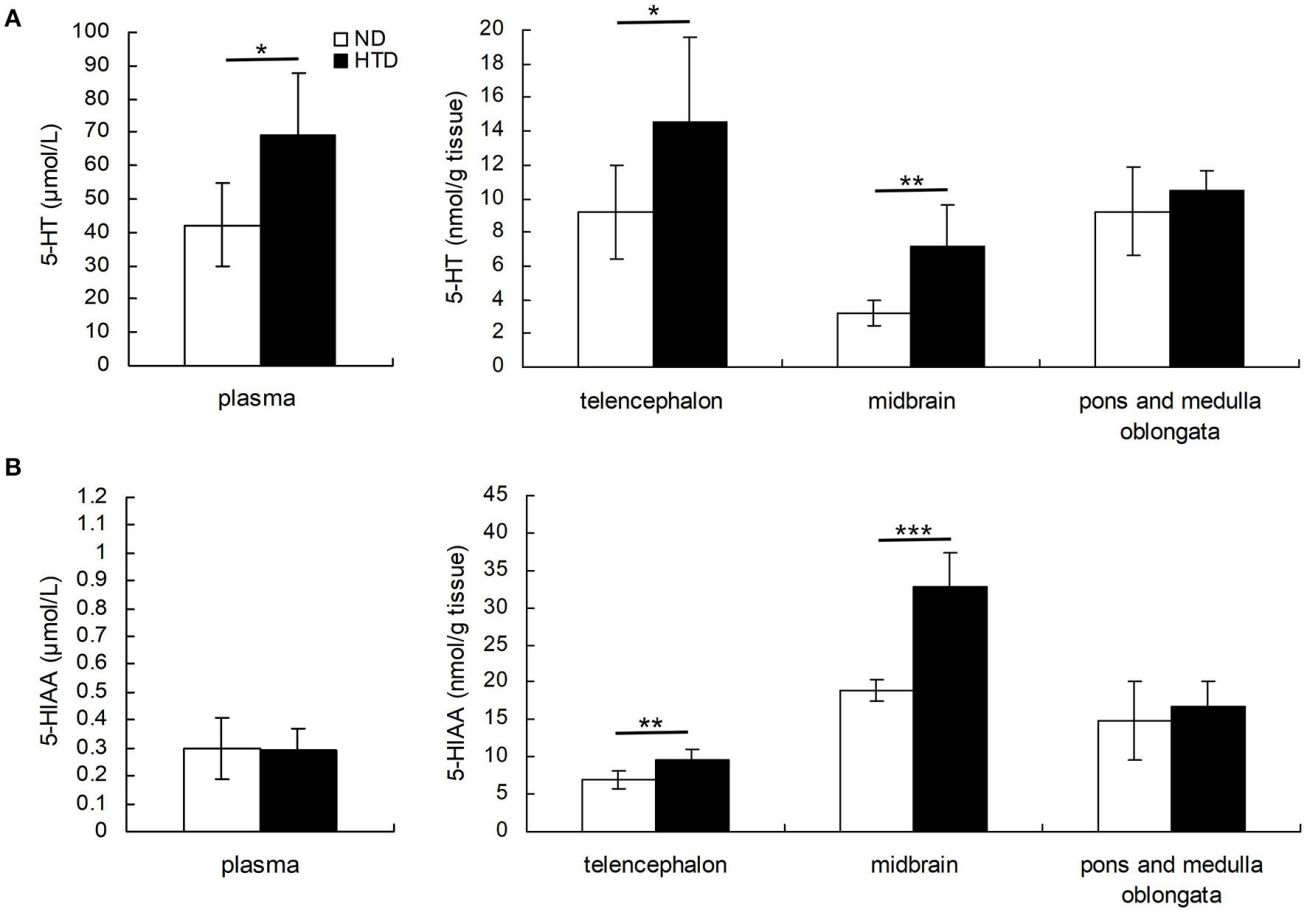
Comparison of the concentrations of 5-HT **(A)** and 5-HIAA **(B)** in plasma and brain regions of DBA/1 mice between the HTD and ND groups. Statistical analysis was performed by the independent samples *t*-test. Data are represented as the mean ± SD. *n* = 6 in each group. *p < 0.05, **p < 0.01, ***p < 0.001. ND, normal diet; HTD, high-tryptophan diet; 5-HT, 5-hydroxytryptamine; 5-HIAA, 5-hydroxyindoleacetic acid.

DBA/1 mice from the HTD group exhibited higher 5-HT levels in the plasma (69.00 ± 18.72 vs. 42.26 ± 12.28, *p* < 0.05), telencephalon (14.56 ± 5.03 vs. 9.21 ± 2.80, *p* < 0.05), and midbrain (7.13 ± 2.46 vs. 3.22 ± 0.71, *p* < 0.01) (Figure 2A). 5-HIAA levels in the telencephalon (9.54 ± 1.36 vs. 6.98 ± 1.15, *p* < 0.01) and midbrain (32.84 ± 4.51 vs. 18.93 ± 1.37, *p* < 0.001) were also higher in DBA/1 mice from the HTD group than in those in the ND group (Figure 2B).

### HTD Altered the Abundance and Diversity of the Gut Microbiota

#### Alpha Diversity

**Figure 3:**
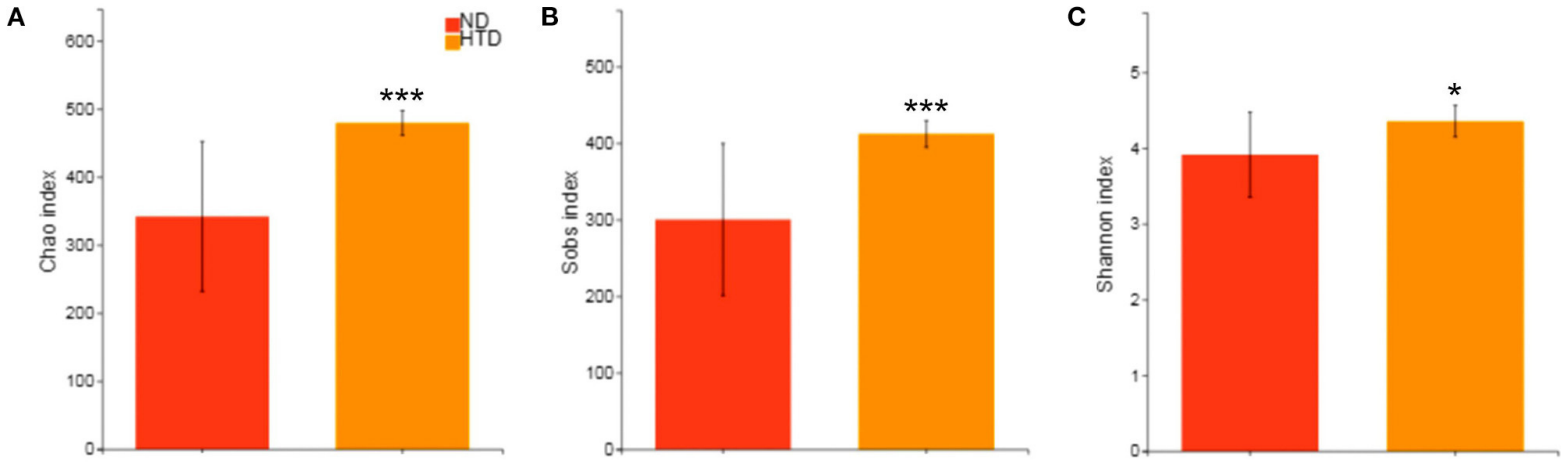
Alpha diversity analyses between the HTD and ND groups. Alpha diversity analysis of the gut microbiota was performed using the Chao estimator **(A)**, Sobs **(B)** and Shannon diversity index **(C)** and performed with the Wilcoxon rank-sum test between the two groups. Data are represented as the mean ± SD. *n* = 10 in each group. *p < 0.05, ***p < 0.001. ND, normal diet; HTD, high-tryptophan diet; Sobs, the observed richness.

The alpha diversity analysis showed significantly higher Chao (479.29 ± 18.02 vs. 341.95 ± 110.18, *p* < 0.001) (Figure 3A), Sobs (412.00 ± 17.39 vs. 300.20 ± 99.27, *p* < 0.001) (Figure 3B), and Shannon (4.36 ± 0.21 vs. 3.91 ± 0.56, *p* < 0.05) indexes (Figure 3C) in the HTD group than in the ND group. This suggests that HTD treatment significantly increased the number of observed OTU sequence tags and the observed richness and species diversity.

#### Beta Diversity

**Figure 4:**
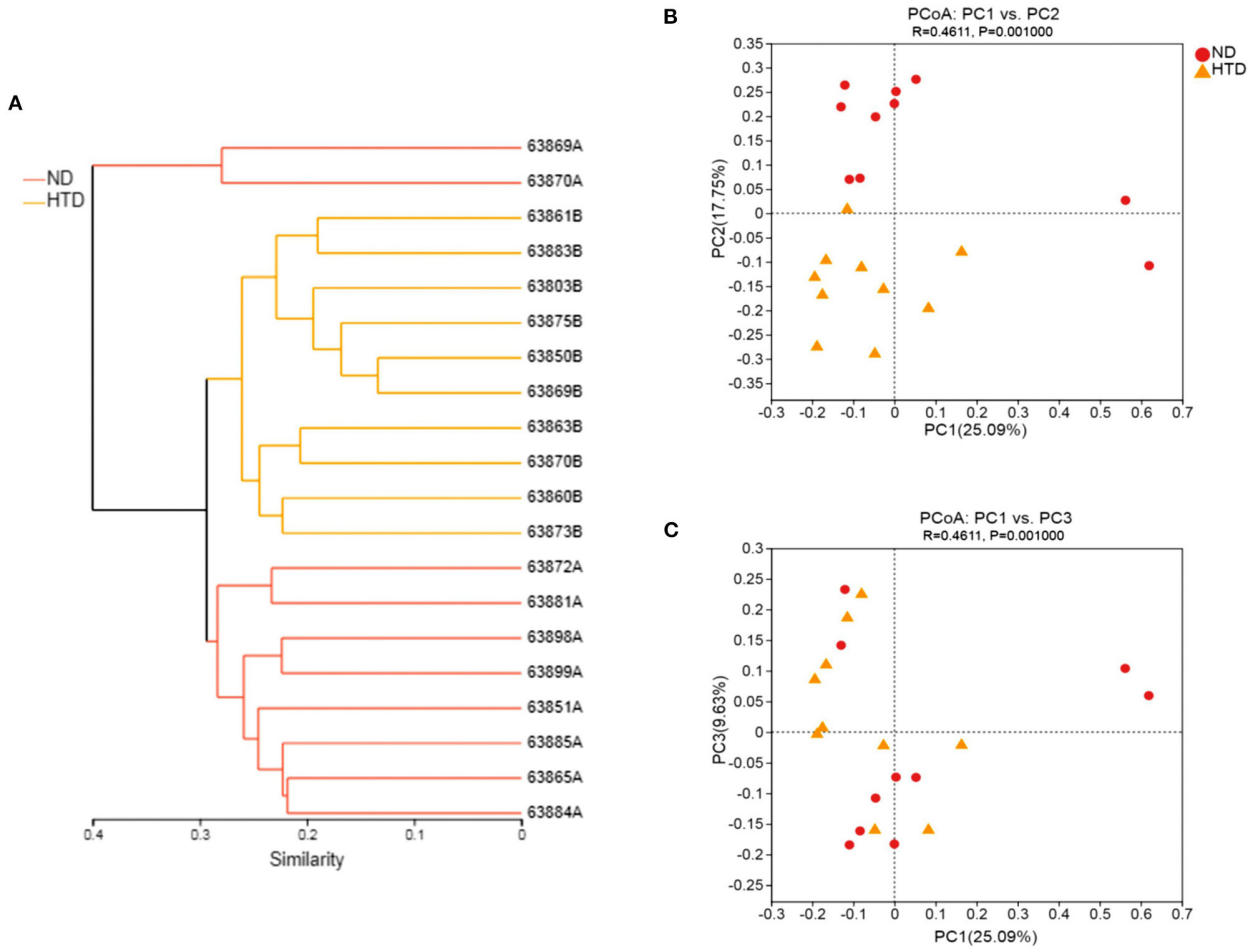
Beta diversity analyses between the HTD and ND groups. **(A)** Hierarchical cluster analysis using Bray-Curtis distances. Each sample was marked with a branch of different color and divided into diverse cohesive groups according to their distance thresholds. **(B,C)** PCoA based on Bray-Curtis dissimilarity. The microbiota of each sample from the ND (red circle) and HTD groups (orange triangle) is represented by different points. *n* = 10 in each group. ND, normal diet; HTD, high-tryptophan diet; PCoA, principal coordinate analysis.

The hierarchical cluster analysis showed that all samples were divided into three distinct subgroups based on fecal bacterial community composition (Figure 4A), indicating that the composition of the bacterial community in the HTD group was markedly different from that of the ND group. PCoA of sequencing data showed significantly separate clustering of the gut microbiota structure between the ND and HTD groups. PC1, PC2, and PC3 accounted for 25.09, 17.75, and 9.63% of the variation, respectively (Figures 4B,C). The microbial composition of the samples from individuals fed with the same diet was similar.

### HTD Altered the Composition of the Gut Microbiota

**Figure 5:**
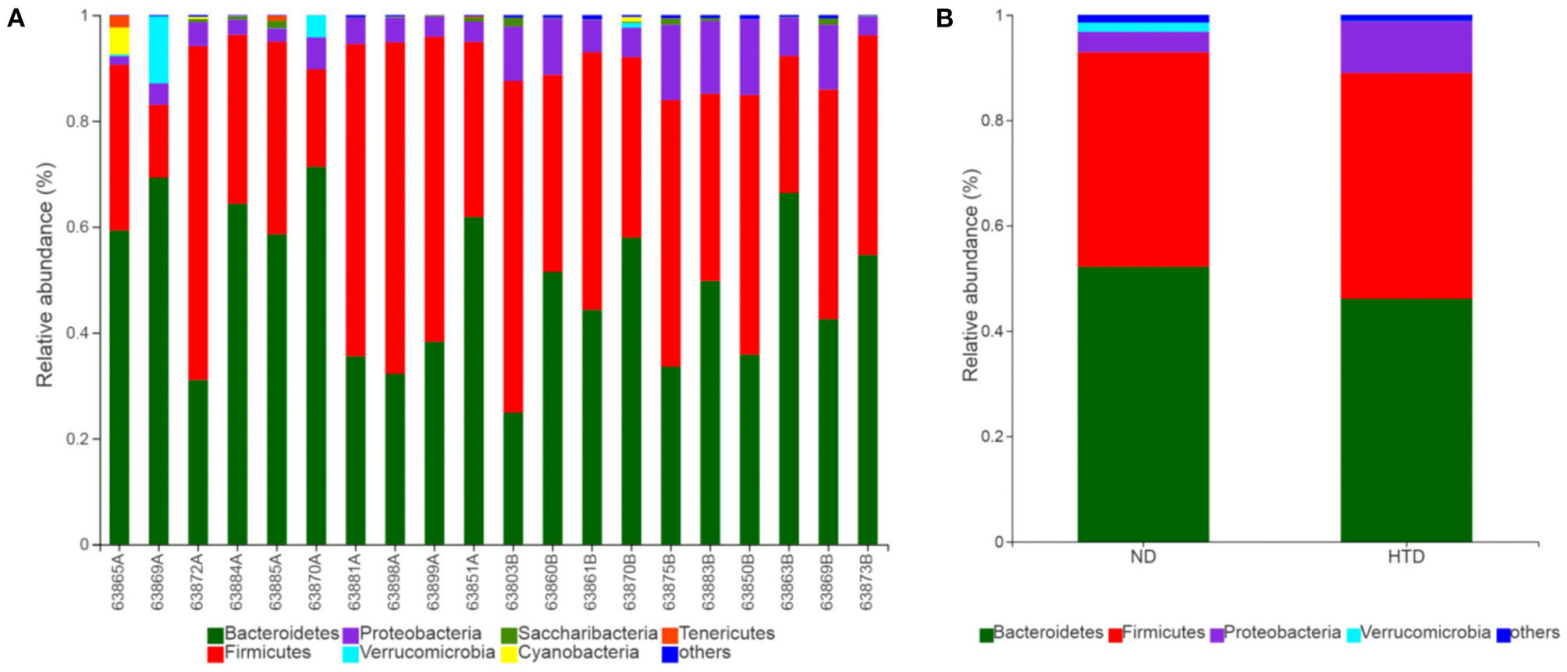
Microbial composition between the ND and HTD groups. Vertical bar charts depict the various species compositions of different samples **(A)** and groups **(B)** at the phylum taxonomic level. *n* = 10 in each group. ND, normal diet; HTD, high-tryptophan diet.

**Figure 6:**
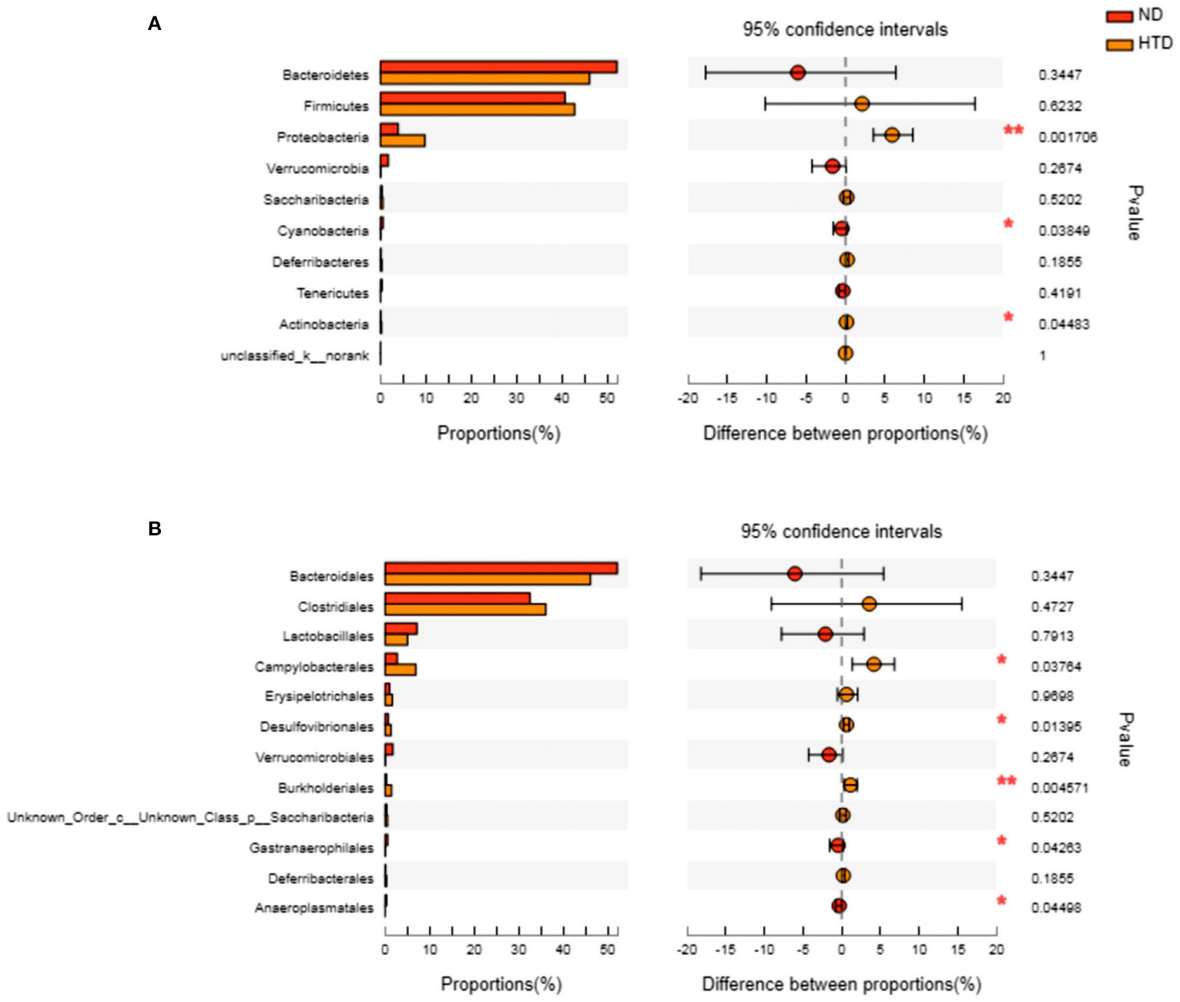
Group differences in the gut microbiota between the ND and HTD groups. The horizontal bar charts depict the taxonomic differences between the two groups at the phylum **(A)** and order **(B)** levels. Statistical analysis was performed by the Wilcoxon rank-sum test. *n* = 10 in each group. *p < 0.05, **p < 0.01. ND, normal diet; HTD, high-tryptophan diet.

As shown in Figures 5A,B, *Bacteroidetes* and *Firmicutes* were the most abundant phyla observed in all samples. At the phylum level, the relative abundances of *Proteobacteria* (*p* < 0.01) and *Actinobacteria* (*p* < 0.05) were significantly increased, and that of *Cyanobacteria* (*p* < 0.05) was strikingly decreased, in the HTD group compared with the ND group (Figure 6A). In addition, at the order level, there was an increase in the relative abundance of *Campylobacterales* (*p* < 0.05), *Desulfovibrionales* (*p* < 0.05), and *Burkholderiales* (*p* < 0.01) and a decrease in the relative abundance of *Gastranaerophilalesa* (*p* < 0.05) and *Anaeroplasmatales* (*p* < 0.05) in the HTD group compared with the ND group (Figure 6B).

## Discussions

Diet therapy as a treatment strategy for epilepsy has a long history. For instance, KD, as a well-known low-carb, high-fat diet, is widely used in the treatment of epilepsy, autism spectrum disorders, and Alzheimer's disease (37, 38). However, to date, effective diet intervention for SUDEP prevention may be limited; although a previous study has reported that S-IRA evoked by acoustic stimulation in DBA/1 mice could be significantly reduced by 5-HTP, a precursor of serotonin synthesis (14). However, 5-HTP was administered intraperitoneally, rather than orally; whether the bioavailability of intraperitoneal administration of 5-HTP is equivalent to that with oral administration is unclear. Alteration of dietary TRP is often used as a non-invasive method to manipulate the TRP levels of the body, thereby affecting 5-HT neurotransmission in the CNS (39). HTD has been applied in the treatment of fatty liver disease (40), diabetes (41), Alzheimer's disease (42), and so on, which indicates that HTD may be safe and feasible as an adjunctive therapy. Our study is the first to demonstrate that HTD is an effective diet intervention in preventing SUDEP in DBA/1 mice.

In this study, we found that an HTD significantly increased 5-HT and 5-HIAA levels in the telencephalon. The previous studies showed that SSRIs significantly increased the 5-HT content in the frontal cortex of rats (43). Extensive synaptic connections were found between the cortex of the telencephalon and 5-HT neurons in the dorsal raphe of the midbrain sublattice (44). In addition, some scholars found that the telencephalon was also involved in the arousal mechanisms of consciousness disorders (45). Therefore, we hypothesized that the increase in telencephalon 5-HT levels may affect S-IRA occurrence in DBA/1 mice through the neural network between the telencephalon and midbrain. We also found that the HTD significantly increased 5-HT and 5-HIAA levels in the midbrain. A previous study showed that the occurrence of S-IRA was significantly inhibited by selectively activating 5-HT neurons in the midbrain through optogenetic technology in transgenic DBA/1 mice (46). We speculated that the HTD-reduced S-IRA in this SUDEP model may due to the elevation of the 5-HT concentration in the midbrain. Interestingly, the 5-HT level was not significantly altered in the pons and medulla of DBA/1 mice after the HTD. In Zhan's study, multiunit recordings showed decreased firing of neuron populations both in the medullary and midbrain raphe, and single-unit recordings of serotonergic neurons revealed consistently decreased firing in the medullary raphe but a mixture of increased and decreased firing in the midbrain raphe during the ictal and postictal periods of an established Sprague Dawley rat seizure model (47). However, the 5-HT level is not equal to 5-HT neuron firing, and neuron firing will increase in one region, thereby leading to 5-HT release in another site (48, 49). In addition, as the literature stated, the midbrain raphe is more likely involved in the mechanism of unconsciousness, and the medullary raphe is considered to be involved in cardiorespiratory dysfunction during and after epileptic seizures (47). In the future, the specific roles of these two nuclei and whether selectively activated 5-HT neurons in the medulla are associated with a reduced incidence of SUDEP should be studied further.

We found that the HTD significantly increased the species abundance and diversity of the gut microbiota compared with ND. In addition, the gut microbiota of the HTD-treated mice was dominated by *Proteobacteria* and *Actinobacteria*. The mechanisms underlying bacterial-induced 5-HT signaling are not well understood. Studies proved that some *Proteobacteria* and *Actinobacteria* species were closely related to the increase in short-chain fatty acid (SCFAs) (50), which were reported to be capable of promoting 5-HT production in peripheral blood (51). Since we did not test the SCFA differences between treatment groups, we did not know if the HTD reduced the S-IRA rate by affecting the gut microbiota and then elevating SCFAs and eventually peripheral and central 5-HT. In addition, some studies found that gut metabolites mediated by certain intestinal flora can regulate brain activity through the autonomic nervous system (52), and the stimulation of peripheral vagal nerves could modulate the concentration of central 5-HT (53). Other bacterial species have also been reported to be capable of regulating 5-HT metabolism. For example, the administration of lipopolysaccharide, a cytoderm component of gram-negative bacteria, significantly increased the production of 5-HT in the prefrontal cortex, striatum, and midbrain of animals (54, 55), possibly via the modulation of TPH activity (55). In addition, some bacterial metabolites, such as acetic acid (an SCFA), can regulate the expression of serotonin receptors in the gut and brain as well as change behaviors in animals (56). Generally, the exact mechanism by which the gut microbiota mediates the changes in 5-HT levels in the CNS through the “gut-brain axis” is relatively complicated and still needs further exploration.

Taking into consideration the important role of central 5-HT synthesis in SUDEP, it is meaningful to detect 5-HT deficiency in patients with epilepsy, which is helpful in differentiating those who are at high risk for SUDEP. Recent studies found that Positron Emission Tomography(PET)/Single-Photon Emission Computed Tomography(SPECT) could monitor alterations of the 5-HT receptor/5-HT transporter in associated brain regions by serotonergic probes (57) or regional blood flow (58) and had been applied in neuropsychiatric and neurodegenerative disorders (59, 60). Therefore, screening high-risk patients with serotonin-targeted Positron Emission Tomography(PET)/Single-Photon Emission Computed Tomography(SPECT) may be a promising strategy for the prevention and treatment of SUDEP in the future.

## Conclusions

To our knowledge, our research was the first to demonstrate that a HTD significantly reduced the incidence of S-IRA and affected the synthesis and metabolism of 5-HT as well as the diversity and composition of the gut microbiota in DBA/1 mice. However, the specific molecular mechanism remains to be further clarified. Our findings may open another window for the pathogenesis of SUDEP, and a HTD is expected to be a promising candidate for the prevention of SUDEP in clinical practice, especially for patients with central serotonin deficiency.

## Data Availability Statement

The datasets presented in this study can be found in online repositories. The names of the repository/repositories and accession number(s) can be found at: https://www.ncbi.nlm.nih.gov/, PRJNA760677.

## Ethics Statement

The animal study was reviewed and approved by the Animal Ethical and Welfare Committee and the Institutional Animal Care and Use Committee, Xiangya Hospital, Central South University, China (No. 202009559).

## Author Contributions

QY and MC carried out experiments and performed the statistical analysis. QY wrote the first draft of the manuscript. BX, CZ, and QZ revised the manuscript. All authors contributed to the study conception and design and critically read and approved the final manuscript.

## Funding

This study was supported by the National Natural Science Foundation of China (CZ: No. 81501130, QZ: No. 81601140, BX: No. 81974206), Natural Science Foundation of Hunan Province, China (QZ: No. 2020JJ5843, CZ: No. 2021JJ31047).

## Conflict of Interest

The authors declare that the research was conducted in the absence of any commercial or financial relationships that could be construed as a potential conflict of interest.

## Publisher's Note

All claims expressed in this article are solely those of the authors and do not necessarily represent those of their affiliated organizations, or those of the publisher, the editors and the reviewers. Any product that may be evaluated in this article, or claim that may be made by its manufacturer, is not guaranteed or endorsed by the publisher.
