# Supplementary material for: A High-Tryptophan Diet Reduces Seizure-Induced Respiratory Arrest and Alters the Gut Microbiota in DBA/1 Mice
Source: Front Neurol. 2021 Nov 23;12:762323. doi: 10.3389/fneur.2021.762323 (PMC8650499; doi:10.3389/fneur.2021.762323)

Supplementary Material

Supplementary Table 1. Nutrients composition of both ND and HTD were similar as shown in the table below. ND, normal diet; HTD, high-tryptophan diet.

Nutrients	Content
**Metabolizable energy**	
Protein	23.07%
Carbohydrate	65.08%
Fat	11.85%
**Vitamin**	
Vitamin A	7800.00 IU/kg
Vitamin D	1200.00 IU/kg
Vitamin E	67.00 mg/kg
Vitamin K	5.00 mg /kg
Vitamin B1	10.00 mg/kg
Vitamin B2	15.00 mg/kg
Vitamin B6	10.00 mg/kg
Vitamin B12	0.02 mg/kg
Nicotinic acid	55.00 mg/kg
Pantothenic acid	22.00 mg/kg
Biotin	0.20 mg/kg
Folic acid	6.60 mg/kg
**Mineral substance**	
Sodium (Na)	3.10 g/kg
Magnesium (Mg)	2.90 g/kg
Kalium (K)	7.40 g/kg
Cuprum (Cu)	11.40 mg/kg
Iron (Fe)	113.70 mg/kg
Manganese (Mn)	80.00 mg/kg
Zinc (Zn)	31.60 mg/kg
Selenium (Se)	0.20 mg/kg
Iodine (I)	0.70 mg/kg

Supplementary Table 2. Aminoacids present in ND and HTD (g/kg). The ND and HTD are the same composition in nutrients except the tryptophan content. ND, normal diet; HTD, high-tryptophan diet.

Aminoacids	ND	HTD
Methionine+Cystine	5.80	5.80
Lysine	8.90	8.90
Arginine	9.90	9.90
Leucine	14.80	14.80
Isoleucine	7.40	7.40
Threonine	6.60	6.60
Valine	8.90	8.90
Histidine	4.90	4.90
Phenylalanine+Tyrosine	14.60	14.60
**Tryptophan**	**2.10**	**4.00**

Supplementary Fig 1. Schematic showing the timeline of seizure induction, diet intervention, acoustic stimulation, UHPLC and 16S rDNA microbiota profiling at various time points in experiment. PND, postnatal day; ND, normal diet; HTD, high-tryptophan diet; UHPLC, ultra-high-pressure liquid chromatography; S-IRA, seizure-induced respiratory arrest.

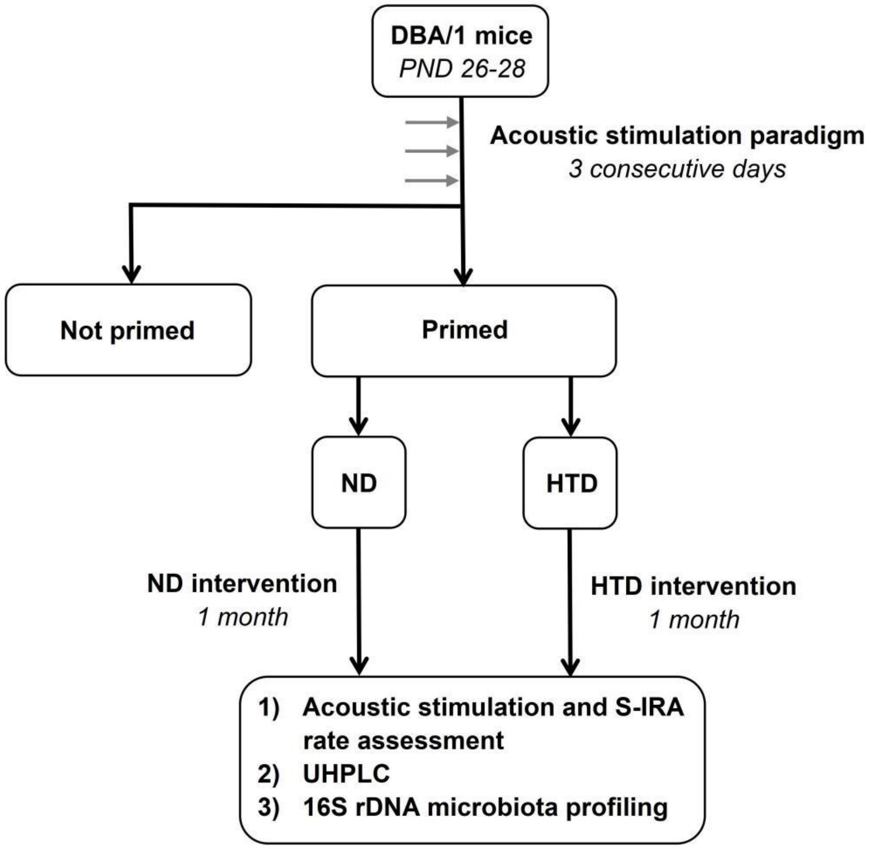

Supplement: Supplementary file 1 [file Presentation_1.pdf]
